# Ribavirin Inhibits the Activity of mTOR/eIF4E, ERK/Mnk1/eIF4E Signaling Pathway and Synergizes with Tyrosine Kinase Inhibitor Imatinib to Impair Bcr-Abl Mediated Proliferation and Apoptosis in Ph+ Leukemia

**DOI:** 10.1371/journal.pone.0136746

**Published:** 2015-08-28

**Authors:** Fangfang Shi, Yamei Len, Yuping Gong, Rui Shi, Xi Yang, Duolan Naren, Tianyou Yan

**Affiliations:** Department of Hematology, West China Hospital, Sichuan University, Chengdu, Sichuan, China; B.C. Cancer Agency, CANADA

## Abstract

The eukaryotic translation initiation factor 4E (eIF4E), which is the main composition factor of eIF4F translation initiation complex, influences the growth of tumor through modulating cap-dependent protein translation. Previous studies reported that ribavirin could suppress eIF4E-controlled translation and reduce the synthesis of onco-proteins. Here, we investigated the anti-leukemic effects of ribavirin alone or in combination with tyrosine kinase inhibitor imatinib in Philadelphia chromosome positive (Ph+) leukemia cell lines SUP-B15 (Ph+ acute lymphoblastic leukemia cell line, Ph+ ALL) and K562 (chronic myelogenous leukemia cell line, CML). Our results showed that ribavirin had anti-proliferation effect; it down-regulated the phosphorylation levels of Akt, mTOR, 4EBP1, and eIF4E proteins in the mTOR/eIF4E signaling pathway, and MEK, ERK, Mnk1 and eIF4E proteins in ERK/Mnk1/eIF4E signaling pathway; reduced the expression of Mcl-1 (a translation substrates of eIF4F translation initiation complex) at protein synthesis level not mRNA transcriptional level; and induced cell apoptosis in both SUP-B15 and K562. 7-Methyl-guanosine cap affinity assay further demonstrated that ribavirin remarkably increased the eIF4E binding to 4EBP1 and decreased the combination of eIF4E with eIF4G, consequently resulting in a major inhibition of eIF4F complex assembly. The combination of ribavirin with imatinib enhanced antileukemic effects mentioned above, indicating that two drugs have synergistic anti-leukemic effect. Consistent with the cell lines, similar results were observed in Ph+ acute lymphoblastic primary leukemic blasts; however, the anti-proliferative role of ribavirin in other types of acute primary leukemic blasts was not obvious, which indicated that the anti-leukemic effect of ribavirin was different in cell lineages.

## Introduction

The eukaryotic translation initiation factor 4E (eIF4E) is over-expressed in many human cancers, such as breast cancer, prostate cancer, and acute myeloid leukemias [1–3]. eIF4E is the main composition factor of eIF4F translation initiation complex, which binds with the 5’7-methyl guanosine (m^7^G) mRNA cap and influences the growth of tumor through modulating cap-dependent protein expression [4]. eIF4E enhances the translation of some regulated onco-proteins, including regulators of cell cycle (CyclinD), apoptosis (Mcl-1), angiogenesis (VEGF), and others. Two main signaling pathways regulate the eIF4E activity, one is the mammalian target of rapamycin (mTOR)/eIF4E-binding proteins (4E-BPs) pathway, and another one is mitogen-activated protein kinase (MAPK)-interacting kinase-1/2 (Mnk1/2) [5,6]. The hypophosphorylated 4EBP1 could prevent the formation of the eIF4F complex by tightly binding with eIF4E to prevent the recruitment of eIF4G, i.e., a scaffolding molecule, to the 5’cap of mRNA. However, the phosphorylation by the mTORC1 (mTOR complex 1) leads to the dissociation of 4EBP1 from eIF4E, allowing for binding of eIF4G and eIF4A to form the eIF4F complex [7]. Thus, PI3K/Akt/mTORC1/eIF4E signaling pathway plays an important role in regulating the protein synthesis. The eIF4E phosphorylation at Ser209 by Mnk1/2 kinases, which are activated by ERK (extracellular regulated protein kinases) and p38 pathway, is also critical for the onco-genic activity of eIF4E [8]. Mnk uses eIF4G as a docking site to phosphorylate eIF4E and strengthens the onco-protein translation function by enhancing the ability of combination with 5’cap structure of mRNA, which promotes tumorigenesis [9,10].

Ribavirin (1-β-D-ribofuranosyl-1,2,4,-triazole-3-carboxamide), a broad-spectrum antiviral drug, physically mimics the m^7^G cap depending protein. Previous studies have shown that ribavirin has antitumor activity in various tumor cells in an eIF4E-dependent manner. Successful ribavirin treatments in the breast cancer and refractory M4/M5 AML patients have attracted great interest along with attentions that ribavirin (eIF4E-targeted agents) treatment could be clinically beneficial in the 30% of cancers characterized by elevated eIF4E with poor prognosis [1,3,11]. A Phase II trial (NCT00559091) demonstrated that targeting eIF4E with ribavirin has significant clinical activity with no treatment-related toxicity in patients with M4/M5 AML [3]. And the combination therapy of ribavirin with some common chemo-therapeutic agents of AML showed a synergistic effect in primary acute myeloid leukemia specimens [11]. Ribavirin has antitumor effect by suppressing eIF4E-controlled translation and inhibiting the synthesis of onco-proteins, including a number of cell growth-related, proliferation-related, and apoptosis-related proteins, such as anti-apoptotic factor Mcl-1, the cell cycle regulators cyclin D1 and D3, pro-vascular endothelial growth factor VEGF, and onco-protein c-Myc. Furthermore, ribavirin impairs eIF4E mediated apoptotic rescue and eIF4E dependent Akt survival signaling by up-regulation of Nijmegen breakage syndrome 1 (NBS1), a Akt pathway activator [4,12–15]. Therefore, it is a promising drug to treat tumor cells with over-expressed eIF4E.

Philadelphia (Ph) chromosome is characterized by the translocation (9;22) (q34;q11) that forms bcr-abl fusion oncogene, which encodes Bcr-Abl fusion protein. Ph chromosome is found in 95% of chronic myelogenous leukemia (CML) and 20–30% of adult acute lymphoblastic leukemia (ALL), which are designated as Ph+ leukemia. Philadelphia chromosome positive acute lymphoblastic leukemia (Ph+ ALL) has poor prognosis when conventional chemotherapy is used. Imatinib, the Bcr-Abl specific tyrosine kinase inhibitor, was developed for CML. It has been widely used in the clinical treatment of CML and has achieved remarkable results [16,17]. Accordingly, many studies have tried to combine imatinib with chemotherapy to treat Ph+ ALL. The results showed that imatinib-combined chemotherapy is effective and feasible for Ph+ ALL and superior to chemotherapy or imatinib alone [18,19]. However, imatinib-resistance leads to the distinct effect in Ph+ ALL, in contrast to CML [20,21]. Hence, the search for new therapeutic approaches for Ph+ ALL is imminent.

Our previous study found that 4EBP1/eIF4E protein translation axis was over-expressed in Ph+ ALL [22]. Bcr-Abl contributed to the phosphorylation of 4E-BP1 (inactivation) and induced the formation of eIF4F translation initiation complex, enhancing the cap-dependent protein expression, in which 4EBP1/eIF4E signaling pathway plays an important role in the pathogenesis of Ph+ leukemia [23]; accordingly, it may be a promising therapeutic target in Ph+ ALL. Therefore, we attempted to use ribavirin to treat eIF4E over-expressed Ph+ ALL.

In this study, Ph+ ALL cell line SUP-B15, CML cell line K562, and primary leukemia blasts were treated with ribavirin alone or in combination with imatinib. The results showed that ribavirin combined with imatinib had obvious synergistic anti-leukemic effect in Ph+ ALL by suppressing the phosphorylation of mTOR/eIF4E signaling pathway and, surprisingly, by inhibiting the ERK/Mnk1/eIF4E signaling pathway, blocking the assembly of eIF4F complexes, and inducing apoptosis. Therefore, ribavirin combined with tyrosine kinase inhibitors might be a new therapeutic option to Ph+ ALL patients in the future.

## Materials and Methods

### Cells and reagents

The human Ph+ ALL cell line SUP-B15 (the American Type Culture Collection, CRL-1929), CML cell line K562 (Hematology Lab, West China Hospital of Sichuan University, Sichuan, China 610041), and primary leukemic blasts were maintained in RPMI 1640 or IMDM (Hyclo, USA), supplemented with 5% penicillin/streptomycin and 10% fetal bovine serum (FBS) in a humidified atmosphere at 37°C in a 5% CO2 incubator. Blast cells were isolated by Ficoll density gradient centrifugation (TBD Science, Tianjin, China). Ribavirin (Sigma, USA) was dissolved in H_2_O at a stock concentration of 20mmol/L and sterile filtered, kept at -80°C in small aliquots. Imatinib (Basel, Switzerland), U0126 (Beyotime), and CGP57380 (Sigma) were prepared in dimethyl sulfoxide (DMSO) stored at -20°C with 10 mmol/L and diluted into suitable concentrations before being used.

### Ethics statement

The bone marrow and peripheral blood samples from 30 acute leukemia or CML patients in the Department of Hematology, the West China Hospital, Sichuan University were obtained after obtaining written informed consent from the patients and approval from the clinical trials and biomedical ethics special committee of West China Hospital of Sichuan University.

### Primary leukemic specimens

The 30 specimens divided into four groups according to the morphologic, immunologic and molecular characteristics of leukemia cells, including 12 cases of Ph+ ALL (9 cases were newly diagnosed, 3 cases were relapsed), 5 cases of Ph-negative ALL (Ph- ALL) (4 cases were newly diagnosed, 1 case was recurrence after allogeneic hematopoietic stem cell transplantation), 5 cases of Ph+ CML (2 cases were blast crisis, 3 case were in the accelerated phase), 8 cases of AML (all cases were newly diagnosed). The relapsed patients of Ph+ ALL and CML have previously accepted the treatment of imatinib.

### Cytotoxicity assay

Cells were plated in RPMI 1640 or IMDM on 96-well plates, with indicated concentrations of ribavirin, imatinib alone, or combination, and incubated at 37°C for 72h. Cytotoxicity was measured using the 3-(4,5-dimethylthiazol-2-yl)-2,5- diphenyltetrazolium bromide (MTT, Sigma) assay and the optical density (OD) quantified with uQuant MQX200 Microplate Spectro-photometer (Biotek) at 570 nm. The cell viability rate was calculated as follows: [OD(agent)−OD(blank)]/[OD(control)−OD(blank)]×100%. IC_50_ (the concentration of drug to kill 50% cells) values were calculated by SPSS17.0, as described previously [24]. The combination index (CI) was used to analyze the synergistic cytotoxicity effect and calculated using the following formula: CI = A_1_/A_m1_+B_2_/B_m2_ (A_1_or B_2_ is the concentration of combined agents that produce the cytotoxicity effect, A_m1_ or B_m2_ is the concentration of single agent to produce the same cytotoxicity effect). CI value less, equal to, or greater than 1 indicated that the interaction of the two drugs is synergistic, additive, or antagonistic, respectively [25].

### Western blotting

Whole cell extracts were prepared by the lysis of cells (1×10^7^) in RIPA lysis buffer (20mM Tris, pH 7.4, 250mM NaCl, 2mM EDTA, pH 8.0, 0.1% Triton-X100, 0.01mg/ml aprotinin, 0.005mg/ml leupeptin, 0.4mM PMSF, 4mM NaVO4) at 4°C and supplemented with protease and phosphatase inhibitors. Total proteins (30–120μg) were loaded onto 6% to 12% sodium dodecyl sulfate (SDS)-polyacrylamide gels and then electro-transferred to polyvinylidene difluoride membranes (Millipore, Billerica, USA). Western blots were performed to detect total Akt, phospho-Akt (Ser473), total mTOR, phospho-mTOR (Ser2448), total 4EBP1, phospho-4EBP1 (Thr37/46), total eIF4E, phospho-eIF4E (Ser209), phospho-cRaf (Ser338), total MEK, phospho-MEK1/2 (Ser217/221), phosphor-ERK1/2 (Thr202/Tyr204), total Mnk1, phospho-Mnk1 (Thr197/202), phospho-Lyn (Tyr396), Mcl-1, and Cyclin D1 (Cell Signaling Technology, USA). The immunoblots were visualized by an enhanced chemiluminescence (ECL) detection system (Bio-Rad Laboratories) according to the manufacturer’s instructions. GAPDH was used to confirm equal protein loading.

### 7-Methyl-guanosine cap affinity assay

Samples (1×10^7^ cells) were lysed in 500μl of RIPA lysis buffer and clarified by centrifugation (13,000g, 20 minutes, 4°C). Aliquots of supernatants were incubated with 50μl of 7^m^-GTP-Sepharose beads (GE Healthcare) for 1 to 2 hours at 4°C. The beads were then washed 3 times in 1ml of phosphate-buffered saline (PBS) and boiled in 50μl of SDS-PAGE sample buffer for 7 minutes. The retained supernatants were analyzed by Western blot with primary antibodies against total 4EBP1, eIF4E, and eIF4G (Cell Signaling Technology, USA), as described in section 5 of the methods section.

### Flow cytometric analysis

SUP-B15 and K562 cells were treated with ribavirin, imatinib, and ribavirin plus imatinib at indicated concentrations for 24h, 48h, and 72h. Apoptotic cells were quantified by flow cytometric analysis with Annexin V-FITC and propidium iodide (PI) double staining using a detection kit (KeyGEN Biotech, Nanjing, China) according to the manufacturer’s instructions.

### Real-time quantitative PCR

SUP-15 and K562 cells were treated with indicated concentration of ribavirin, imatinib, and ribavirin plus imatinib for 48h. Total RNA extraction, reverse transcription, and real-time polymerase chain reaction (PCR) were carried out as previously described [26]. Forward primer of *Mcl-1* is CCA GGC AAG TCA TAG AAT and reverse primer is GAG GCT TAC AGT CAT AGT T while GAPDH forward primer is GTG AAC CAT GAG AAG TAT GAC AAC and reverse primer is CAT GAG TCC TTC CAC GAT ACC.

## Results

### 1. The anti-proliferation effects of ribavirin alone or in combination with imatinib on SUP-B15 and K562 cell lines

**Fig 1:**
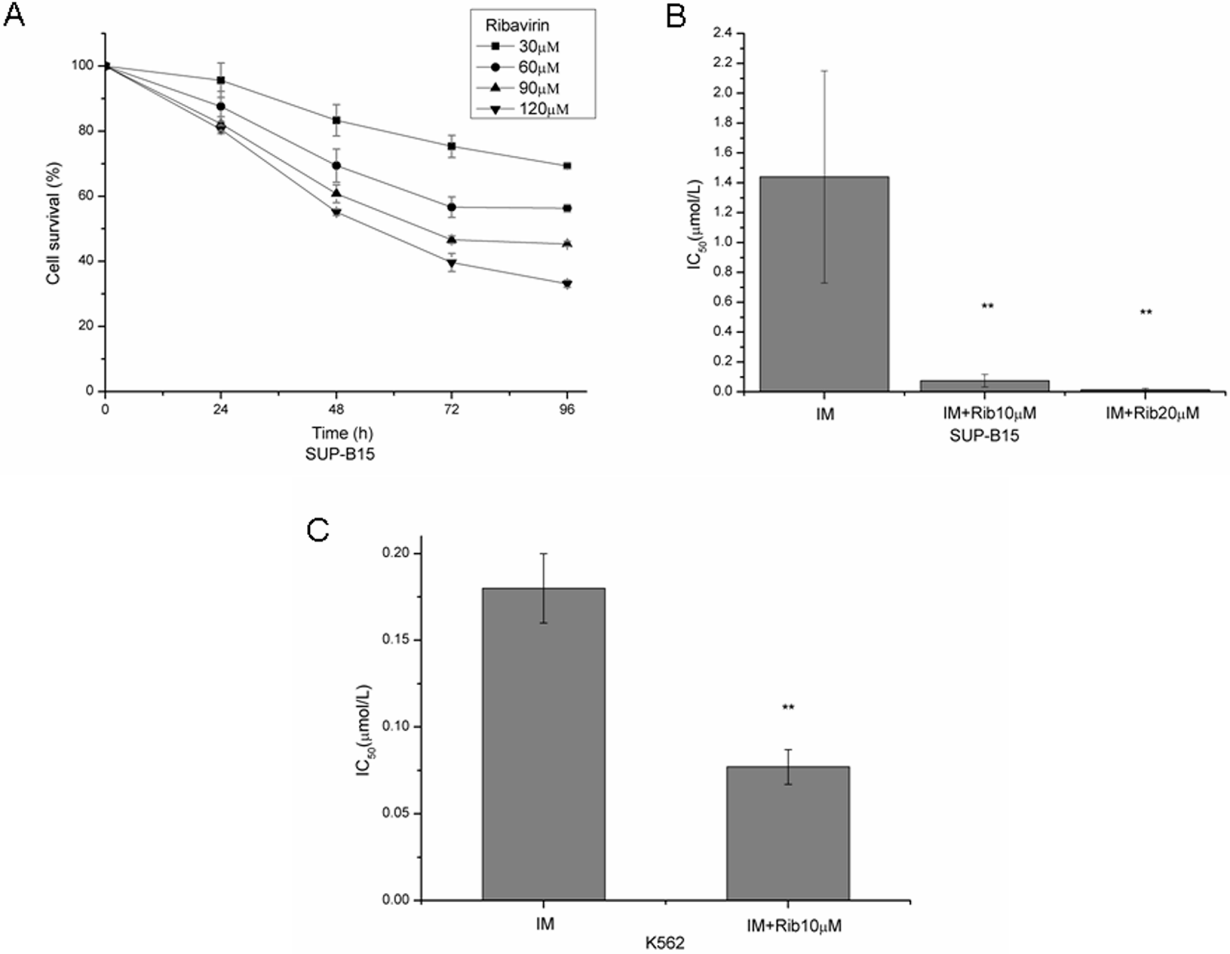
Anti-leukemic effect of ribavirin alone or combined with imatinib in Ph+ leukemia cell lines. A. SUP-B15 cells were treated with 30, 60, 90, and 120μM ribavirin for 24, 48, 72, and 96h. Cell proliferation was assessed by the MTT assay and cell survival rates were presented. B. SUP-B15 cells were treated with a series of concentrations of imatinib (0.001–20μM) alone or combined with 10μM or 20μM ribavirin for 72h, the IC_50_ values of imatinib were shown. C. K562 cells were treated with 1μM imatinib or combined with 10μM ribavirin for 72h, the IC_50_ values of imatinib were shown. The data represent means±SD of three experiments. * represents p<0.05, ** represents p<0.01.

Previous studies have shown that myelogenous leukemia cells are sensitive to ribavirin [3,15]. Here, we examined the effect of ribavirin on the viability in Ph+ leukemia cell lines SUP-B15 and K562. The results showed that ribavirin significantly inhibited the proliferation of SUP-B15 in a dose-dependent and time-dependent manner (Fig 1A). The same results were found for K562 cells (S1 Fig). After 72 hours exposure, the IC_50_ values of ribavirin and imatinib alone were 65.18±11.8μM and 1.44±0.71μM for SUP-B15, and 78.30±5.16μM and 0.18±0.02μM for K562 cells, respectively. However, combined with 10μM or 20μM ribavirin, the IC_50_ values of imatinib decreased to 0.075±0.043μM and 0.015±0.009μM in SUP-B15 cell line (Fig 1B). The differences between imatinib alone and imatinib plus ribavirin were statistically significant (p = 0.005 and p = 0.004, respectively). The combination index (CI) calculated for 50% inhibition of cell growth was 0.206 and 0.317, which indicated that the two drugs had synergistic effect on anti-proliferation. Consistently, the IC_50_ value of imatinib combined with 10μM ribavirin in the K562 cell lines was 0.077±0.01μM (Fig 1C) with the CI of 0.556. These results indicated that the combination of imatinib and ribavirin had synergistic anti-leukemic effect in Ph+ leukemia cell lines.

### 2. Ribavirin inhibited the signaling pathways of mTOR/eIF4E, MEK/ERK/Mnk1/eIF4E in SUP-B15 and K562 cell lines

**Fig 2:**
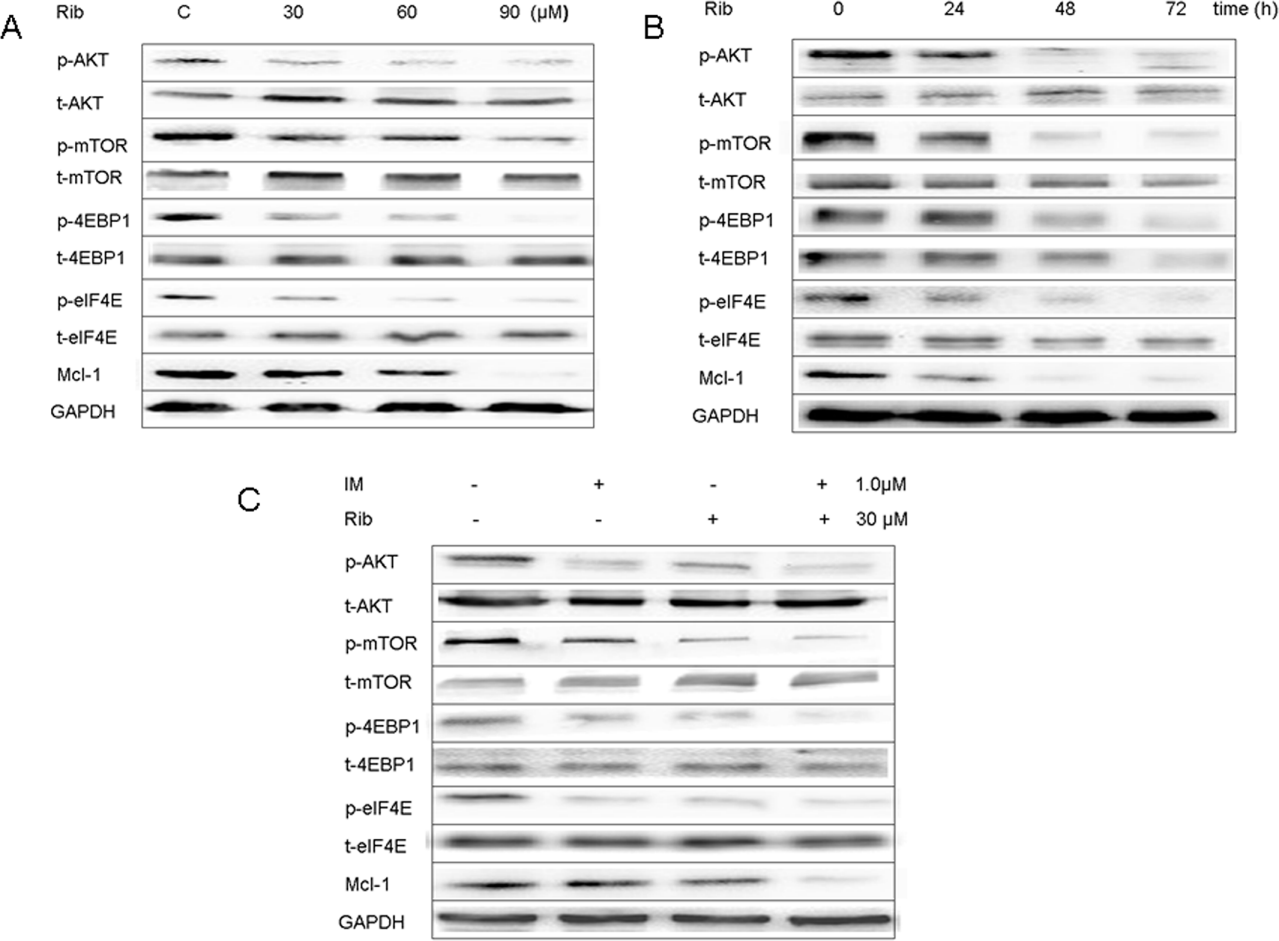
Ribavirin inhibited the signaling pathway of mTOR/eIF4E in SUP-B15. A. The SUP-B15 cells were treated with different concentrations of ribavirin for 48h, and the proteins in mTOR/eIF4E signaling pathway and Mcl-1 were detected by western bolt analysis. B. The SUP-B15 cells were incubated in 60μM ribavirin for 24, 48, 72h, and the mTOR/eIF4E pathway expression was detected by western bolt analysis. C. The expression of mTOR/eIF4E pathway and Mcl-1 in SUP-B15 cells after treated with ribavirin (30μM), imatinib (10μM) alone, and 30μM ribavirin plus 10μM imatinib for 48h.

To study the anti-leukemic mechanisms of ribavirin at the protein level, western blot analysis was used to investigate the expression of mTOR/eIF4E signaling pathways after ribavirin treatment. The results showed that 30–90μM ribavirin down-regulated the phosphorylation levels of mTOR at Ser2448, 4EBP1 at Thr37/46, and eIF4E at Ser209 proteins in the mTOR/eIF4E signaling pathway, resulting in the reduction of the translation substrates Mcl-1 in dose and time-responsive manners in SUP-B15 cell line (Fig 2A and 2B). Meanwhile, ribavirin strongly inhibited the phosphorylation of Akt at Ser473, as reported previously [11–13]. The combination of ribavirin with imatinib down-regulated the phosphorylation level of these proteins more significantly than ribavirin alone (Fig 2C). The same results were found in K562 cell line (S2 Fig).

**Fig 3:**
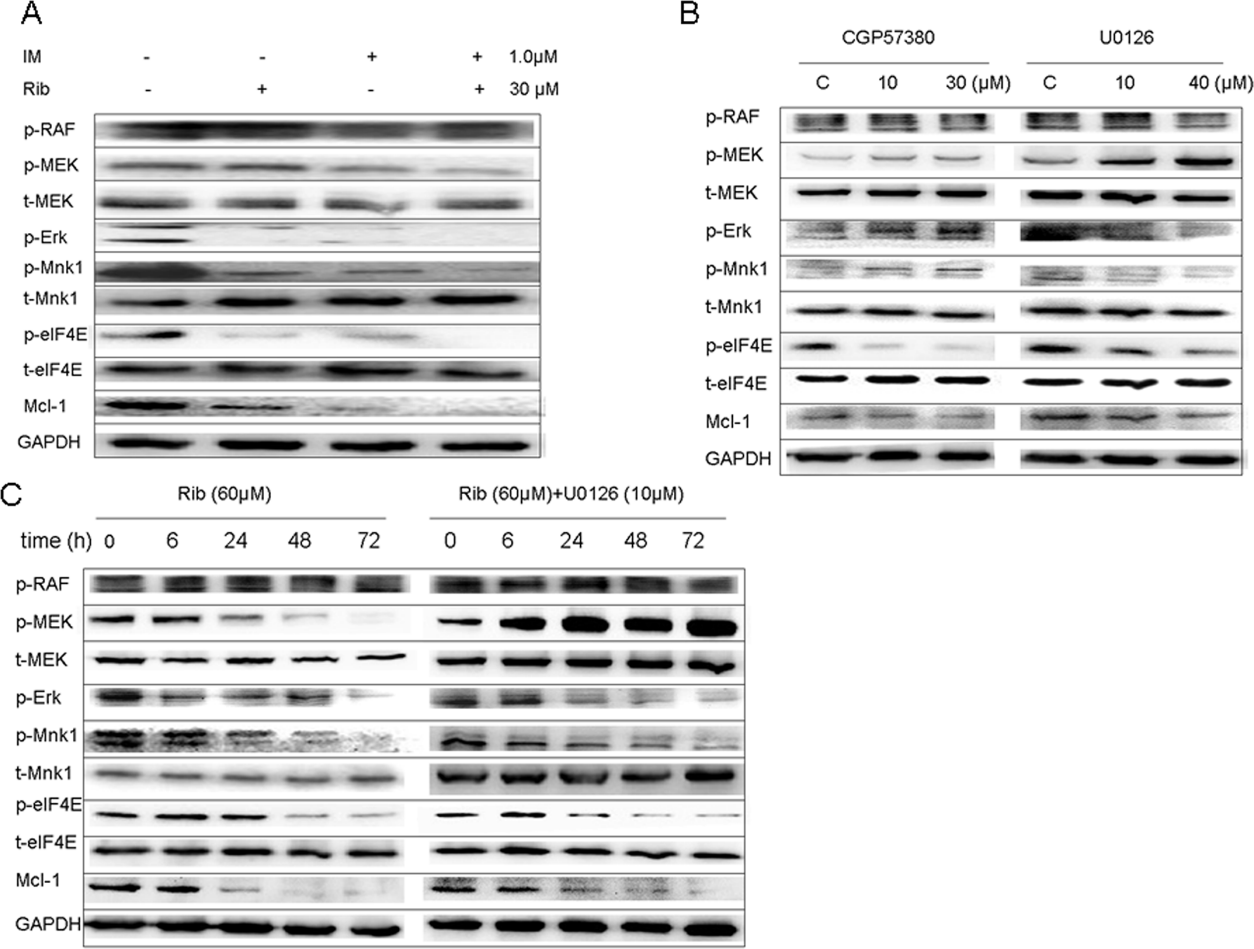
Ribavirin inhibited the MEK/ERK/Mnk1/eIF4E signaling pathway in SUP-B15. A. The expression of MEK/ERK/Mnk1/eIF4E pathway and Mcl-1 in SUP-B15 cells was detected after treated with ribavirin (30μM), imatinib (10μM) alone, and 30μM ribavirin plus10μM imatinib for 48h. B. Indicated concentrates of U0126 (MEK1/2 inhibitor) or CGP57380 (Mnk1 inhibitor) alone treated SUP-B15 cells and the MEK/ERK/Mnk1/ eIF4E signaling pathway expression were analyzed. C. The SUP-B15 cells were treated with 60μM ribavirin alone or plus 10μM U0126 (MEK1/2 inhibitor) for 6, 24, 48, and 72h, and the expression of MEK/ERK/Mnk1/eIF4E pathway and Mcl-1 was detected by western bolt analysis.

Meanwhile, we investigated the effect of ribavirin on the MEK/Mnk1/eIF4E signaling. Similarly, ribavirin down-regulated the phosphorylation levels of MEK at Ser217/221, ERK at Thr202/Tyr204, Mnk1 at Thr197/202, and eIF4E at Ser209 proteins in ERK/Mnk1/eIF4E signaling pathway. Moreover, the results indicated ribavirin acted at MEK level. The combination of ribavirin with imatinib down-regulated the phosphorylation level of these proteins more significantly than did ribavirin alone (Fig 3A). Further, SUP-B15 cells were incubated with MEK1/2 inhibitors U0126 and Mnk1 inhibitor CGP57380 or in combination with ribavirin. As expected, U0126 effectively inhibited the phosphorylation of ERK1/2, Mnk1, and eIF4E; thus, the function of MEK was inhibited. Similarly, CGP57380 blocked the phosphorylation of eIF4E (the substrates of Mnk1), indicating that the function of Mnk1 was inhibited (Fig 3B). The combination of ribavirin and U0126 down-regulated the phosphorylation level of these proteins more significantly compared to ribavirin alone (Fig 3C).

These results confirmed that ribavirin could repress the eIF4E function through down-regulating the phosphorylation of mTOR, MEK/Mnk1/2 signaling pathways, reducing the onco-protein synthesis, and having an anti-leukemic role in Ph+ leukemia cell lines.

### 3. EIF4F complex formation is inhibited by ribavirin in SUP-B15 and K562 cell lines

**Fig 4:**
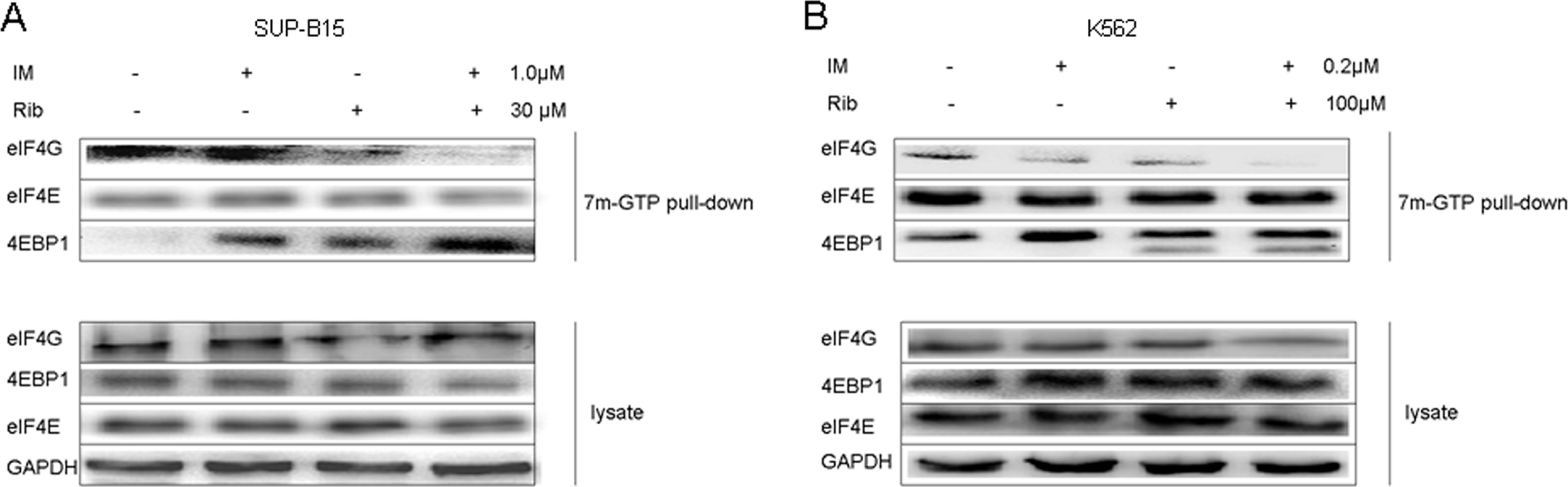
eIF4F complex formation is inhibited by ribavirin in SUP-B15 and K562 cell lines. A. The SUP-15 cells were treated with ribavirin, imatinib alone, or ribavirin plus imatinib for 48h. 1×10^7^ cells were lysed in 500μl of RIPA lysis buffer. 7^m^GTP-Sepharose beads were added into part of supernatants, incubated for 1–2h, and solubilized in 50μl of SDS-PAGE sample buffer. The buffer was boiled for 7 min and was immunoblotted with the primary antibodies against eIF4G, eIF4E, and 4EBP1. The retained supernatants were analyzed by western blot with primary antibodies against eIF4G, eIF4E, 4E-BP1, and GAPDH, as control. B. The K562 cells were conducted by 7^m^GTP pull-down analysis, like SUP-B15 cells.

As described previously, eIF4E-controlled translation is closely relevant to the formation of eIF4F complex. Therefore, we investigated the effect of ribavirin on eIF4F assembly with the pull-down assays using 7^m^-GTP-Sepharose beads, which mimic the cap structure of mRNA [27]. SUP-B15 and K562 leukemia cells were treated separately with ribavirin, imatinib, and ribavirin plus imatinib for 48h. The results of western blot analysis showed that the two drugs did not change the expressions of 4EBP1, eIF4E, and eIF4G in whole cell lysis. However, after 7^m^-GTP pull-down, ribavirin increased the combination of eIF4E and 4EBP1 while it decreased the combination of eIF4E and eIF4G in both SUP-B15 and K562 cells; therefore, it inhibited the assembly of eIF4F translation initiation complex. When combined with imatinib, the inhibition effect on eIF4F translation initiation complex was more obvious; moreover, the change extent was more apparent in SUP-B15 cells than in K562 cells (Fig 4).

### 4. The effect of ribavirin, imatinib alone, and their combination on the expression of *Mcl-1* mRNA and protein in SUP-B15 and K562 cell lines

Mcl-1 is an anti-apoptotic protein, and the results above indicated that ribavirin down-regulated the expression of Mcl-1 on protein level. To evaluate whether ribavirin influences Mcl-1 mRNA level, the real-time quantitative PCR was performed to examine the level of *Mcl-1* mRNA expression after being incubated with the indicated concentration of ribavirin, imatinib, ribavirin plus imatinib for 48h in SUP-B15 and K562 cell lines. The results showed that the expression of Mcl-1 did not change at the mRNA level in both cell lines (p>0.05) (S3A and S3C Fig) but changed at protein levels (S3B and S3D Fig), which indicated that ribavirin affects Mcl-1 expression at protein but not at mRNA level.

### 5. The effect of ribavirin and imatinib on the apoptosis in SUP-B15 and K562 cell lines

**Fig 5:**
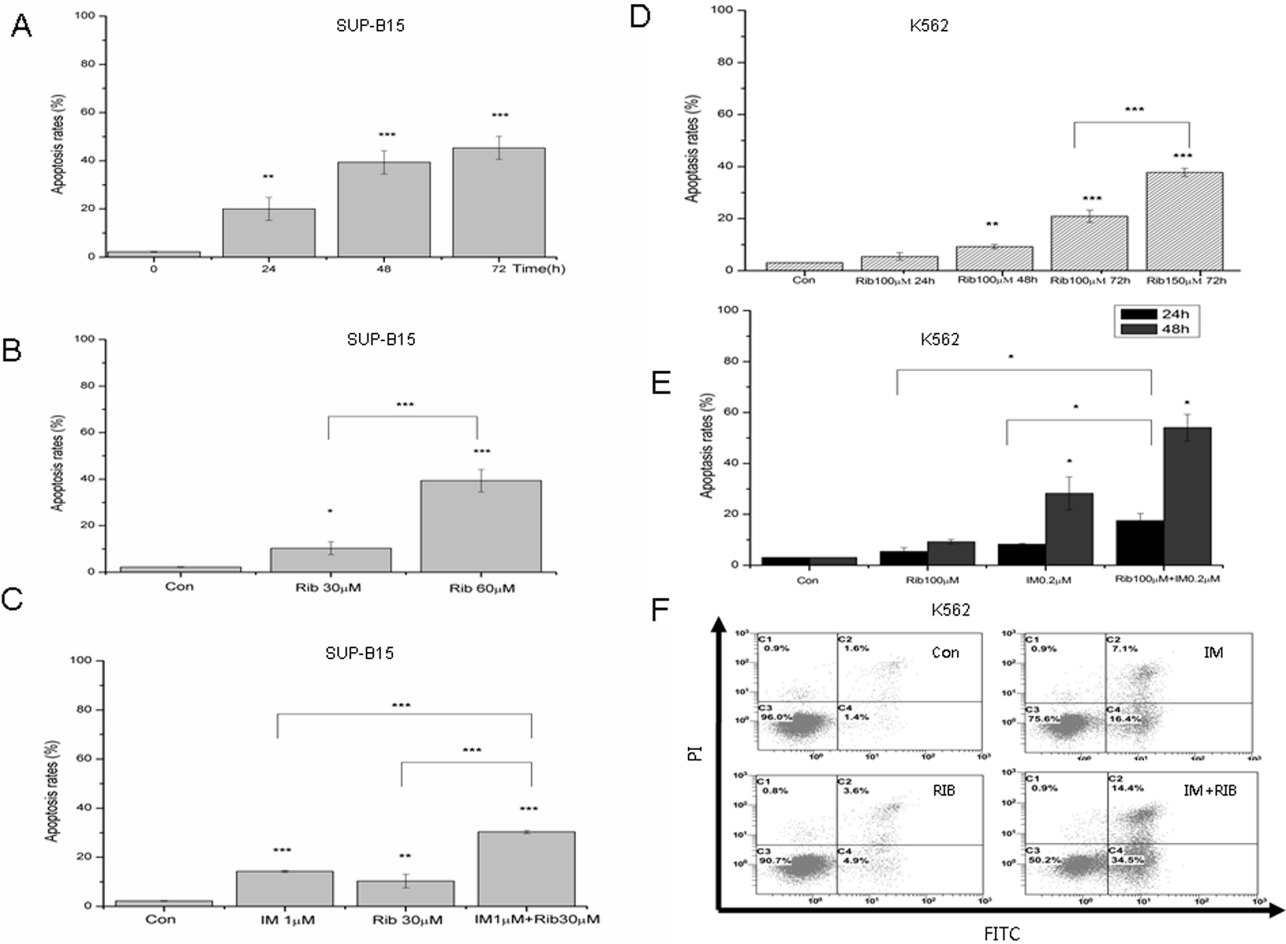
The effect of ribavirin on apoptosis rates in SUP-B15 and K562 cell lines. A. SUP-B15 cells were cultured with ribavirin at the 60μM for 24, 48, and 72h, and apoptosis rates were assessed by flow cytometry after staining of the cells with annexin V-FITC and PI, PBS was used as a negative control. B. The apoptosis rates of SUP-B15 cells when cultured with ribavirin at 30 and 60μM for 48h. C. The apoptosis rates of SUP-B15 cells when cultured with1μM imatinib, 30μM ribavirin alone, or combined for 48h. D. K562 cells were cultured with ribavirin at the indicated doses for 24, 48 and 72h, and apoptosis rates were assessed by flow cytometry after staining of the cells with annexin V-FITC and PI. E. The apoptosis rates of K562 cells when treated with 100μM ribavirin alone or combined with 0.2μM imatinib for 24 or 48h. F. K562 cells were treated with 100μM ribavirin, 0.2μM imatinib alone or combined for 48h, and apoptosis rates were assessed by flow cytometry after staining of the cells with annexin V-FITC and PI. * represents p<0.05, ** represents p<0.01, *** represents p<0.001.

Next, the effect of ribavirin on apoptosis rates was tested with flow cytometry (FCM) in SUP-B15 and K562 cell lines. SUP-B15 and K562 cells were incubated with indicated concentrations of ribavirin, 1μM, or 0.2μM imatinib, and ribavirin plus imatinib for 24h, 48h, and 72h. The apoptotic rates were detected by FCM (Annexin V-FITC double staining). Compared with 2.3% of apoptotic rate in control, the apoptotic rate of SUP-B15 cells increased to 10.36% and 36.0% after the exposure to 30 and 60μM ribavirin for 48h, respectively. The apoptotic rate increased to 18.4%, 36.0%, and 45.4% following treatment with 60μM ribavirin for 24, 48, and 72h, respectively, in SUP-B15 cells. Imatinib alone had a moderate pro-apoptosis effect on SUP-B15, and apoptosis rate was 13.8%. However, 30μM ribavirin plus imatinib resulted in an increase in apoptotic rate to 31.1% (48h) (Fig 5A–5C). As shown in Fig 5D and 5E, ribavirin induced the apoptosis in a time and dose-dependent manner in K562 cells, and the combination of imatinib with ribavirin exerted a significant synergistic effect. Together, these results suggested that ribavirin induced apoptosis with a time and dose-dependent response and had a synergistic effect with imatinib in both SUP-B15 and K562 cell lines. The induction of apoptosis maybe a key action for ribavirin-induced cell death in Ph+ leukemia cells.

### 6. Ribavirin played an anti-leukemic role via mTOR/eIF4E, ERK/Mnk1/eIF4E signaling pathways in Ph+ ALL primary blasts

**Fig 6:**
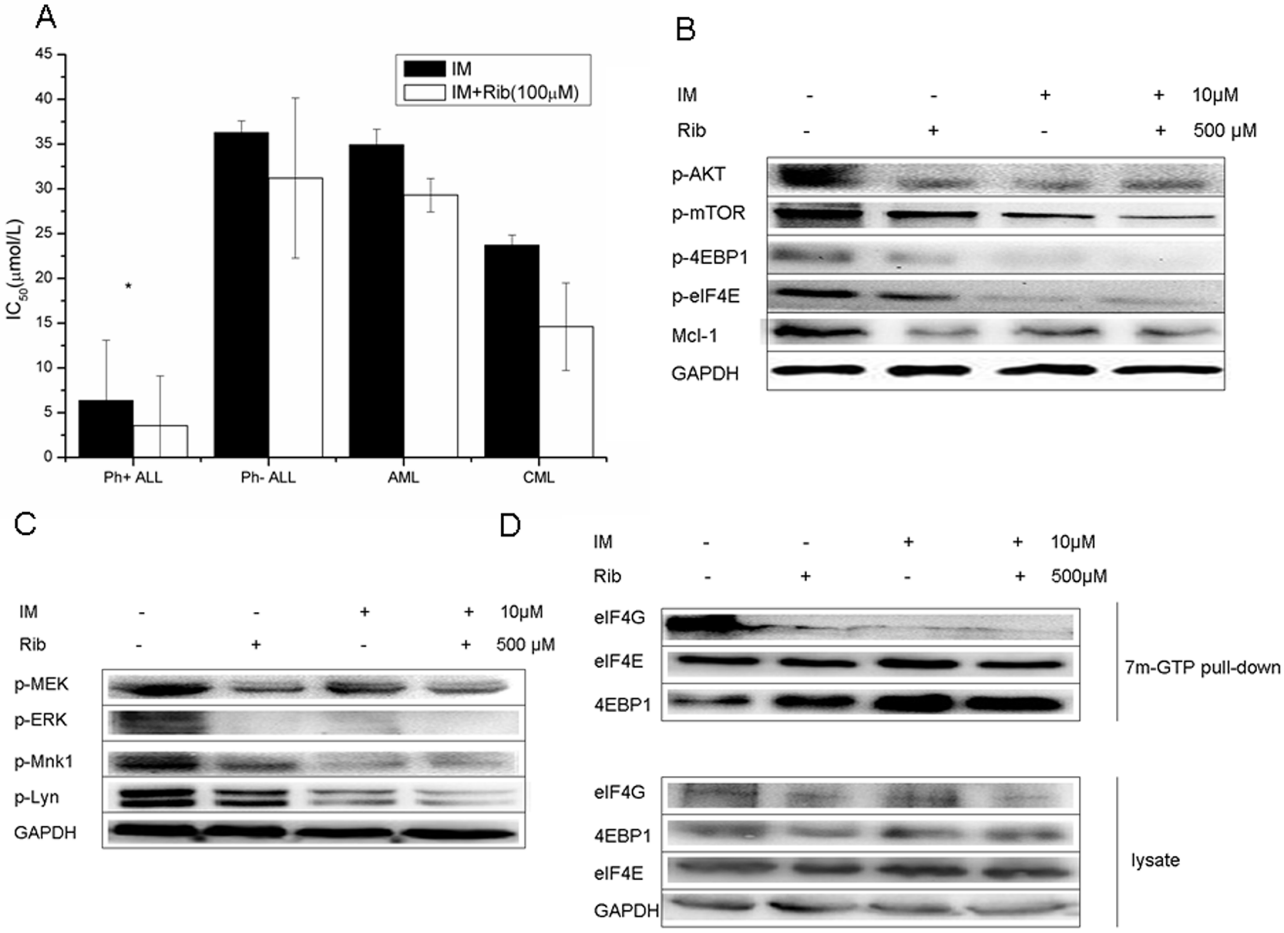
Ribavirin suppressed the cell growth and activation of mTOR/eIF4E, ERK/Mnk1/eIF4E signaling pathways in Ph+ ALL primary blasts. A. The primary leukemia bone marrow and peripheral blood samples were collected and treated at a series of concentrations of imatinib alone or combined with 100μM ribavirin. The MTT assay was performed and IC_50_ values of imatinb alone or in combination were calculated by SPSS17.0. The differences of IC_50_ values between imatinib alone and combination with ribavirin in four groups (Ph+ ALL, Ph–ALL, AML, CML) were analyzed by two paired sample t-tests, * represents p<0.05. B. The primary leukemia blasts were treated with 10μM imatinib, 500μM ribavirin, or combination, and the whole cell lysate was analyzed by western blot with the indicated antibodies, PBS was used as a negative control. The expression of mTOR/eIF4E signaling pathway in one of Ph+ ALL primary blasts was shown. C. The expression of ERK/Mnk1/eIF4E signaling pathways in the primary blasts from one Ph+ ALL patient. D. The 7^m^GTP pull-down analysis was performed in the primary blasts from one Ph+ ALL patient and the expressions of 4EBP1, eIF4E, and eIF4G in 7^m^-GTP pull down or whole cell lysate were exhibited.

In the next study, anti-leukemic role of ribavirin was investigated in primary leukemic blasts. The blasts were treated with ribavirin, imatinib alone, or combination of both. MTT assay, western blot analysis, and 7-Methyl-guanosine cap affinity assay were performed. The results of MTT assay showed that ribavirin alone had a moderate anti-proliferation effect in Ph+ ALL primary blasts with the average inhibition rate of 57.8% in 1mM ribavirin. However, it had little inhibition effect on cell growth in Ph- acute lymphoblastic leukemia and Ph+ CML, with the average inhibition rates of 26.8% and 29.9%, respectively, at the same concentration. The differences between these groups and group Ph+ ALL were statistically significant, with p values of 0.003 and 0.002, respectively. Ribavirin at low concentration (100μM) enhanced the inhibition of imatinib to Ph+ ALL blasts. The mean IC_50_ of imatinib alone was 6.37μM and decreased to 3.56μM when combined with 100μM ribavirin. The difference was statistically significant (p = 0.039). In contrast, the same concentrations of ribavirin did not have a significant effect on the anti-proliferation of imatinib in other leukemia primary blasts, including Ph-ALL (p = 0.152), AML (0.177), and CML (p = 0.147) (Fig 6A).

Western blot analysis was performed in 11 cases to test the effect of ribavirin on growth-signaling pathways when the number of primary blasts was enough (more than 1×10^7^). Blast cells were treated with 10μM imatinib, 500μM ribavirin, and combination for 48h, and the results suggested that ribavirin alone significantly reduced the phosphorylation of mTOR/eIF4E, ERK/Mnk1/eIF4E signaling pathways, substrates Mcl-1, and p-Lyn in all 6 cases of Ph+ ALL primary blasts, and the combination of ribavirin and imatinib had greater effect on the signaling pathways, as described in the cell lines (Fig 6B and 6C). However, the effect of ribavirin on down-regulating the phosphorylation of these signaling pathways in other leukemia primary blasts was not obvious (S4 Fig), which indicated that the anti-leukemic effect of ribavirin was difference in different cell lineages.

One case of Ph+ ALL blasts were incubated with ribavirin (500μM), imatinib (10μM), and ribavirin plus imatinib for 48h, and 7-Methyl-guanosine cap affinity assay was executed. The results showed that ribavirin and imatinib alone remarkably increased the eIF4E binding to 4EBP1 and decreased the combination of eIF4E with eIF4G, consequently resulting in a major inhibition of eIF4F complex assembly. The combination of two drugs had a stronger inhibition effect (Fig 6D).

## Discussion

Previous reporters have shown that ribavirin could be clinically beneficial as antitumor agent in patients with poor AML prognosis characterized by elevated eIF4E with no significant treatment-related toxicity [3]. Furthermore, aberrant cap-dependent mRNA translation may be a therapeutic target in Bcr-Abl-driven malignancies [23]. Therefore, we come up with combining ribavirin and imatinib to treat Ph+ ALL.

Our results demonstrated that ribavirin alone had dose- and time-dependent anti-proliferative effects and that the combination of imatinib and ribavirin inhibited the cell growth synergistically in Ph+ ALL and CML cell lines. Ribavirin had a moderate growth-inhibition effect and significantly enhanced the anti-leukemic effect of imatinib in Ph+ ALL primary blasts; however, little growth-inhibition or promotion effects were observed in the other acute leukemia and CML blasts. What would be the possible reasons of this phenomenon? Hence, we carried out the next study to explore the mechanism of ribavirin’s anti-leukemia on Ph+ acute lymphoblastic leukemia. The precise mechanism by which eIF4E induces onco-genic transformation remains unclear, but some evidence suggests that the Akt/mTOR and ERK/Mnk1 pathways regulate the activity of eIF4E [23,25,28]. eIF4E is an important target for oncogenic translational deregulation in PI3K/AKT/mTOR and RAS/MEK signaling pathways, which act on the downstream eIF4E, affecting the expression of onco-protein and apoptosis proteins [6,23]. Hence, we investigated the effect of ribavirin on those two signal pathways. The results showed that ribavirin alone down-regulated the phosphorylation levels of Akt, mTOR, 4EBP1, and eIF4E proteins in the Akt/mTOR/eIF4E signaling pathway as well as MEK, ERK, Mnk1, and eIF4E proteins in the ERK/Mnk1/eIF4E signaling pathway in both Ph+ cell lines SUP-B15 and K562. Since the phosphorylation of eIF4E on Ser209 regulated by Mnk1 in the ERK/Mnk1/eIF4E pathway plays an important role in proliferation and eIF4E-eIF4G binding, we further investigated the effect of this signaling pathway on the phosphorylation of eIF4E on Ser209. Our data showed that ribavirin suppressed the phosphorylation of eIF4E by MEK/ERK/Mnk1/eIF4E pathway, moreover, the target site of ribavirin was located at the MEK level with little inhibition effect on p-Raf, and ribavirin combined with MEK inhibitor U0126 blocked the eIF4E phosphorylation more significantly than did ribavirin alone. Accordingly, U0126 alone induced the phosphorylation of MEK (upstream of p-ERK) and CGP57380 up-regulated the phosphorylation levels of ERK and Mnk1 (upstream of p-eIF4E), which are considered negative-feedback loops [28–30]. Hence, we concluded that ribavirin inhibited the phosphorylation of 4EBP1 by Akt/mTOR/eIF4E pathway and the phosphorylation of Mnk1 by ERK/Mnk1 pathway down-regulated the eIF4E phosphorylation levels, thereby blocking the assembly of eIF4F and interrupting protein translation, which maybe a crucial anti-leukemic mechanism of ribavirin.

The Akt/mTOR and ERK/Mnk1 pathway were also essential downstream signaling pathways for Bcr-Abl-mediated transformation. In theory, imatinib, which is the Bcr-Abl inhibitor, could block the Bcr-Abl-mediating signaling, and imatinib plus ribavirin should show a prominent anti-leukemic effect. As expected, the combination of ribavirin and imatinib down-regulated the phosphorylation level of those proteins in the Akt/mTOR and ERK/Mnk1 pathways in the SUP-B15 and K562 cell lines, especially p-eIF4E, and the effect was more significantly than that of ribavirin alone. Additionally, consistent results were observed in all 4 cases of Ph+ ALL primary blasts while the effects on the other types of acute leukemia and CML primary blasts were uncertain when using the same concentrations of ribavirin. Our previous study found that the main activated pathway was Raf/MEK/ERK signaling in K562 cells while the Akt/mTOR/eIF4E and Raf/MEK/ERK signaling pathways were both highly activated in SUP-B15 cells. The activation levels of Akt/mTOR/4EBP1 in SUP-B15 cells were markedly higher than in K562 cells [25]. Furthermore, it has been reported that ribavirin can impair eIF4E through NBS1/Akt pathway [1,12]. As stated above, ribavirin had anti-leukemic effects by inhibiting activation of Akt/mTOR/4EBP1 and MEK/ERK/Mnk/eIF4E pathways. Accordingly, we supposed that the anti-leukemic effect of ribavirin was more effective in SUP-B15 cell line maybe because it inhibited both signaling pathways simultaneously while relatively single pathway target in K562 cell line; hence, its effect was not so significant. Of course, we still cannot exclude the precise cause due to the few number of primary blast samples. It was easy to speculate that the other types of acute leukemia with negative Bcr-Abl expression have worse consequences when performing combined treatment with imatinib and ribavirin because of the lack of target point. We observed that imatinib even up-regulated the mTOR/4EBP1/eIF4E axis in some primary Ph- ALL cases (S4B Fig).

We further found that the eIF4F translation is of major importance in Ph+ acute lymphoblastic leukemia after the ribavirin treatment through 7-Methyl-guanosine cap affinity assay. As mentioned above, ribavirin is a physical mimic of the 5′7-methyl guanosine cap structure, competing with endogenous mRNAs for binding with eIF4E [4,6]. The formation of eIF4F complex was strongly inhibited after the exposure to ribavirin for 48 hours, as expected. Surprisingly, imatinib alone also exerted similar results. When treating cells with ribavirin and imatinib simultaneously, the inhibition activity was dramatically superior to the effects of ribavirin or imatinib alone in SUP-B15, while we cannot see such a big difference in K562. When tested with the two drugs alone in 1 case of Ph+ ALL primary sample, the increasing of the 4EBP1 levels and decreasing amounts of eIF4G bound to eIF4E were detected, and the effect was more obvious when exposed to the two drugs together. These results indicated that ribavirin inhibited the eIF4E assembly and that the combination of ribavirin and imatinib could fully inhibit the formation of initial translation complex in SUP-B15 than K562 cells, resulting in the decrease of cap-dependent translation. Maybe this is another reason that ribavirin combined with imatinib exerted an extraordinary anti-leukemic effect on Ph+ ALL patients.

Mcl-1 associated with the pro-apoptotic Bcl-2 family protein Bim contributed to cell survival [31]. Mcl-1 is a downstream of eIF4F mRNA translation that depends highly on eIF4E [6]. Inhibition expression of Mcl-1 induces apoptosis of tumor cells [9,10,32]. Therefore, Mcl-1 will be an important therapeutic target of antitumor in the future. In the present study, our results exhibited that ribavirin inhibited the Mcl-1 synthesis at protein translation not mRNA transcriptional level in Ph+ leukemia cell lines.

In summary, our studies demonstrated that ribavirin down-regulated the phosphorylation level of mTOR/eIF4E and ERK/Mnk1/eIF4E signaling pathways, inhibited the assembly of eIF4F translation initiation complexes, and reduced translation of onco-protein Mcl-1; therefore, it inhibited the proliferation of Ph+ ALL, CML cell lines and primary Ph+ acute lymphoblastic leukemic blasts. More importantly, ribavirin, combined with imatinib, exerted a strong synergistic anti-leukemic role in Ph+ ALL. So we assume ribavirin combining with imatinib maybe a novel therapy to Ph+ ALL.

## Supporting Information

S1 Fig Ribavirin inhibited the proliferation of K562 cells in a dose-dependent and time-dependent manner. K562 cells were treated with 0, 10, 50, 100μM ribavirin for 72h and 100μM for 96h. Cell proliferation was assessed by the MTT assay and cell survival rates were presented.
(TIF)

S2 Fig Ribavirin inhibited the signaling pathways of mTOR/eIF4E and MEK/ERK/Mnk1/eIF4E in K562 cells. A. The K562 cells were incubated with 100μM ribavirin for 24, 48, 72h, and the expression of mTOR/eIF4E signaling pathway was detected by western bolt analysis. B. The K562 cells were treated with a series of concentrations of ribavirin (0, 50, 100, 150μM) for 48h, and the proteins expression of mTOR/eIF4E signaling pathway and Mcl-1 were detected by western bolt analysis. C. The expression of mTOR/eIF4E signaling pathway and Mcl-1 in K562 cells after treated with ribavirin (100μM), imatinib (0.2μM) alone, or 100μM ribavirin plus 0.2μM imatinib for 48h. D. The expression of MEK/ERK/Mnk1/eIF4E signaling pathway and Mcl-1 in K562 cells was detected after treated with ribavirin (100μM), imatinib (0.2μM) alone, or 100μM ribavirin plus 0.2μM imatinib for 48h.
(TIF)

S3 Fig The effect of ribavirin, imatinib on the expression of Mcl-1 mRNA and protein in SUP-B15 and K562 cell lines. A. The expression level of Mcl-1 mRNA in SUP-B15 cells was detected by real-time quantitative PCR after incubated with ribavirin (30μM), imatinib (1μM), and ribavirin (30μM) plus imatinib (1μM) for 48h. B. The expression level of Mcl-1 protein in SUP-B15 was detected by Western Blots after incubated with ribavirin (30μM), imatinib (1μM), and ribavirin (30μM) plus imatinib (1μM) for 48h. The indicated relative density of Mcl-1 to GAPDH in single blot measured by QUANTITY ONE software (Version 4.6.2) was shown below the figures. C. The expression level of Mcl-1 mRNA in K562 was detected by real-time quantitative PCR after incubated with ribavirin (100μM), imatinib (0.2μM), and ribavirin (100μM) plus imatinib (0.2μM) for 48h. D. The expression level of Mcl-1 protein in K562 was detected by western blots after incubation with ribavirin (100μM), imatinib (0.2μM), and ribavirin (100μM) plus imatinib (0.2μM) for 48h. The indicated relative density of Mcl-1 to GAPDH was shown.
(TIF)

S4 Fig The effect of ribavirin on the mTOR/eIF4E, ERK signaling pathways in CML and Ph- ALL primary blasts. The primary leukemia blasts were treated with 10μM imatinib, 500μM ribavirin, or combination, and the whole cell lysate was analyzed by western blot with the indicated antibodies, PBS was used as a negative control. A. The expression of mTOR/eIF4E signaling pathway and p-ERK in one of CML blast crisis patient was shown. B. The expression of mTOR/eIF4E signaling pathway in one patient of Ph- ALL primary blasts.
(TIF)
