# Supplementary figures and images for: Ribavirin Inhibits the Activity of mTOR/eIF4E, ERK/Mnk1/eIF4E Signaling Pathway and Synergizes with Tyrosine Kinase Inhibitor Imatinib to Impair Bcr-Abl Mediated Proliferation and Apoptosis in Ph+ Leukemia
Source: PLoS One. 2015 Aug 28;10(8):e0136746. doi: 10.1371/journal.pone.0136746 (PMC4552648; doi:10.1371/journal.pone.0136746)

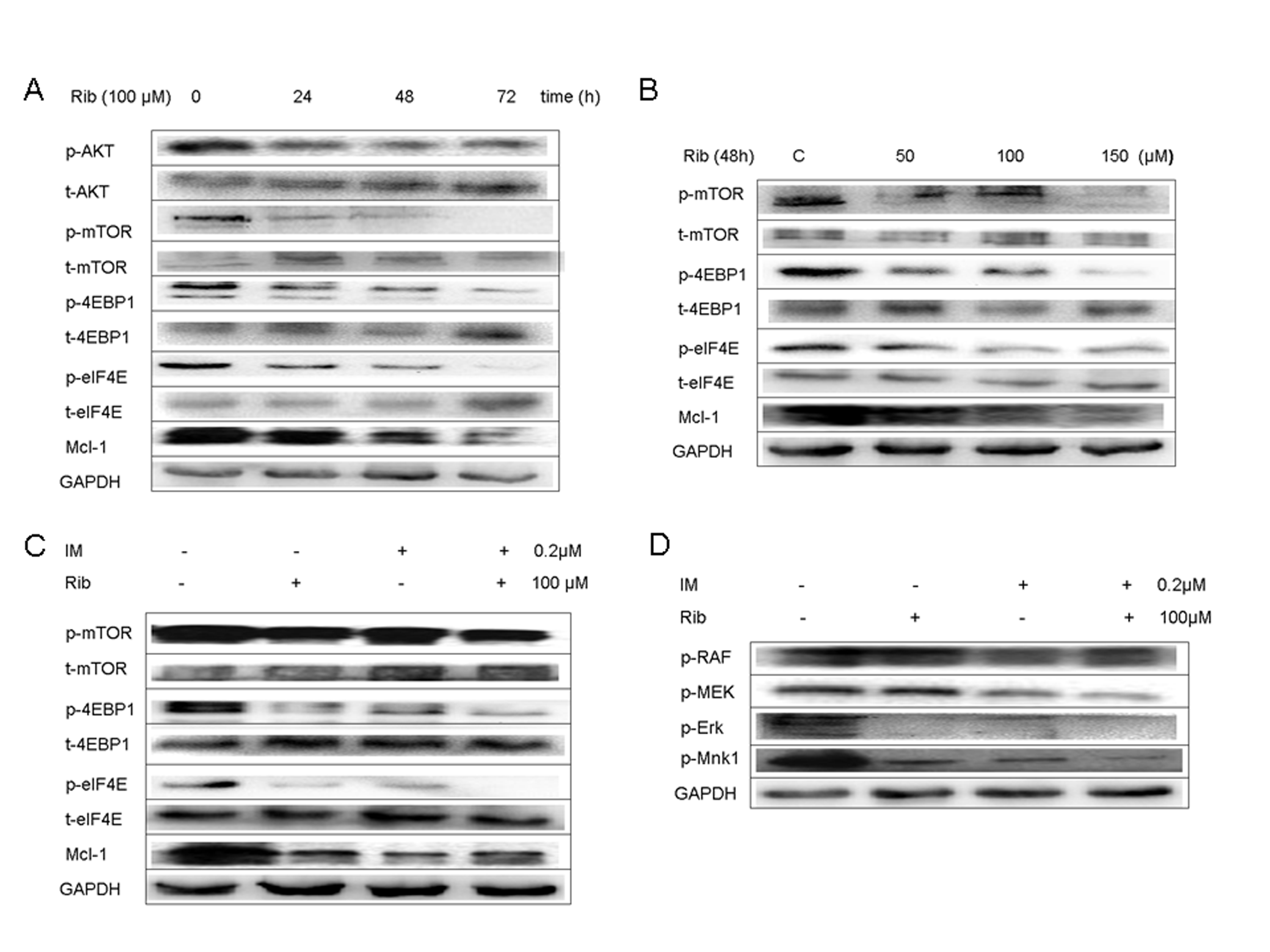

Supplement: S2 Fig — A. The K562 cells were incubated with 100μM ribavirin for 24, 48, 72h, and the expression of mTOR/eIF4E signaling pathway was detected by western bolt analysis. B. The K562 cells were treated with a series of concentrations of ribavirin (0, 50, 100, 150μM) for 48h, and the proteins expression of mTOR/eIF4E signaling pathway and Mcl-1 were detected by western bolt analysis. C. The expression of mTOR/eIF4E signaling pathway and Mcl-1 in K562 cells after treated with ribavirin (100μM), imatinib (0.2μM) alone, or 100μM ribavirin plus 0.2μM imatinib for 48h. D. The expression of MEK/ERK/Mnk1/eIF4E signaling pathway and Mcl-1 in K562 cells was detected after treated with ribavirin (100μM), imatinib (0.2μM) alone, or 100μM ribavirin plus 0.2μM imatinib for 48h. (TIF) [file pone.0136746.s002.tif]

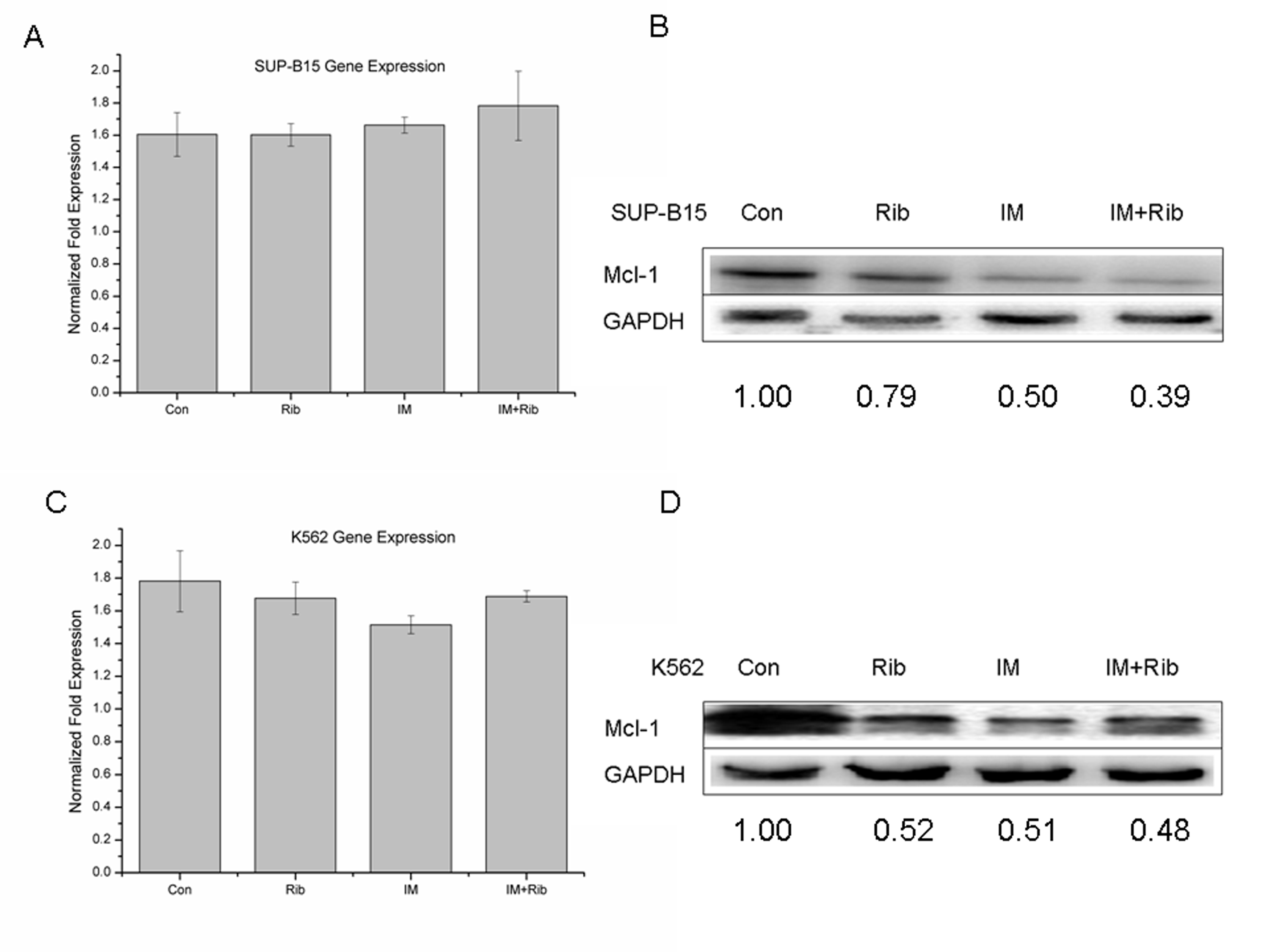

Supplement: S3 Fig — A. The expression level of Mcl-1 mRNA in SUP-B15 cells was detected by real-time quantitative PCR after incubated with ribavirin (30μM), imatinib (1μM), and ribavirin (30μM) plus imatinib (1μM) for 48h. B. The expression level of Mcl-1 protein in SUP-B15 was detected by Western Blots after incubated with ribavirin (30μM), imatinib (1μM), and ribavirin (30μM) plus imatinib (1μM) for 48h. The indicated relative density of Mcl-1 to GAPDH in single blot measured by QUANTITY ONE software (Version 4.6.2) was shown below the figures. C. The expression level of Mcl-1 mRNA in K562 was detected by real-time quantitative PCR after incubated with ribavirin (100μM), imatinib (0.2μM), and ribavirin (100μM) plus imatinib (0.2μM) for 48h. D. The expression level of Mcl-1 protein in K562 was detected by western blots after incubation with ribavirin (100μM), imatinib (0.2μM), and ribavirin (100μM) plus imatinib (0.2μM) for 48h. The indicated relative density of Mcl-1 to GAPDH was shown. (TIF) [file pone.0136746.s003.tif]

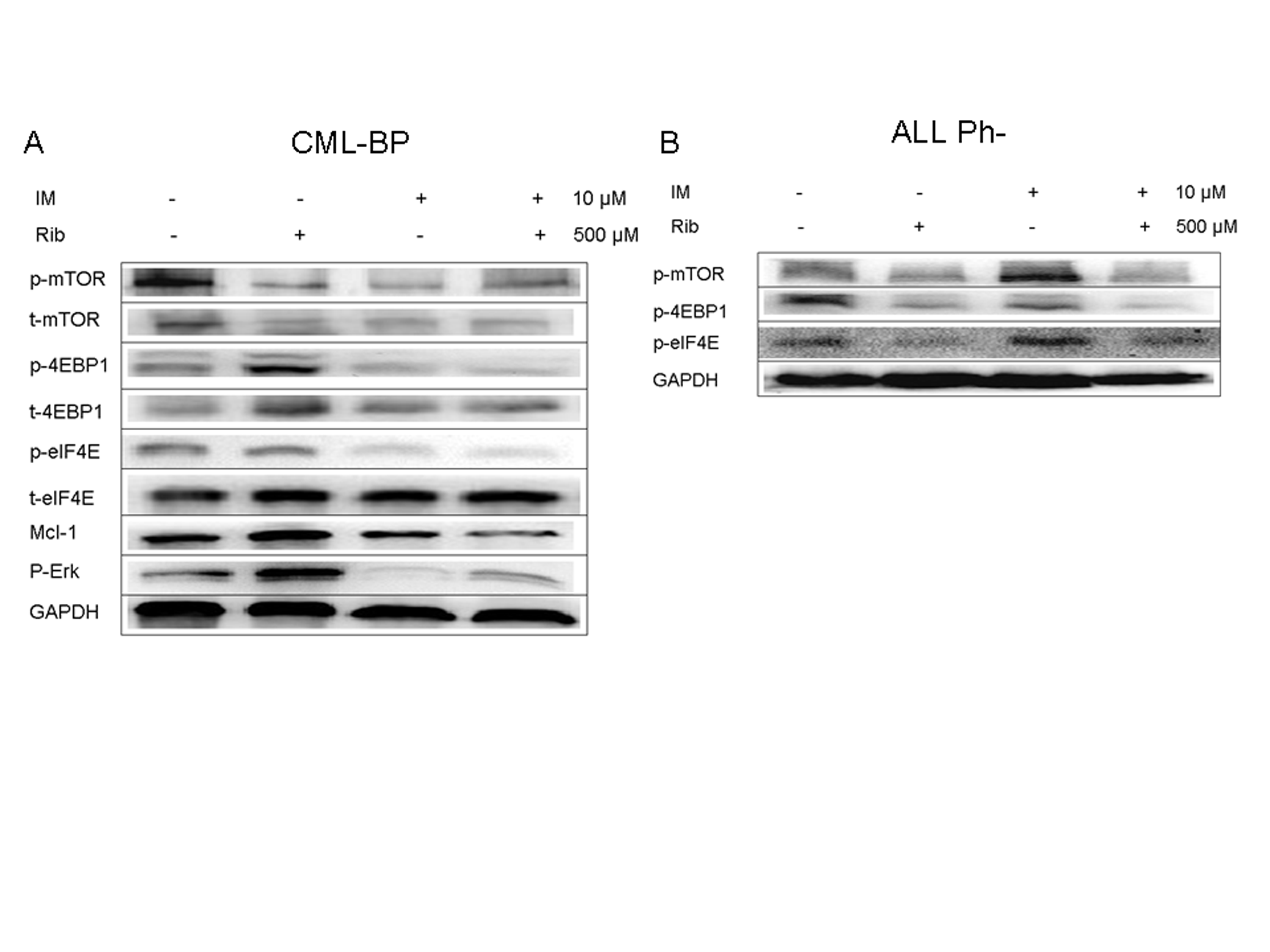

Supplement: S4 Fig — The primary leukemia blasts were treated with 10μM imatinib, 500μM ribavirin, or combination, and the whole cell lysate was analyzed by western blot with the indicated antibodies, PBS was used as a negative control. A. The expression of mTOR/eIF4E signaling pathway and p-ERK in one of CML blast crisis patient was shown. B. The expression of mTOR/eIF4E signaling pathway in one patient of Ph- ALL primary blasts. (TIF) [file pone.0136746.s004.tif]
